# *catena*-Poly[[bis­(dimethyl sulfoxide-κ*O*)cerium(III)]tris­(μ_2_-3,5-dinitro­benzoato-κ^2^ *O*:*O*′)]

**DOI:** 10.1107/S1600536811015947

**Published:** 2011-05-07

**Authors:** Fu-Lin Zhou, Zhi-Hua Du, Seik Weng Ng

**Affiliations:** a Department of Applied Chemistry, College of Chemistry and Life Science, Yuncheng University, Yuncheng, Shanxi 044000, People’s Republic of China; b Plant Protection and Plant Quarrantine Station, Agriculture and Animal Husbandry Bureau, Wulanchabu, Inner Mongolia 012000, People’s Republic of China; c Department of Chemistry, University of Malaya, 50603 Kuala Lumpur, Malaysia

## Abstract

The polymeric title compound, [Ce(C_7_H_3_N_2_O_6_)_3_(C_2_H_6_OS)_2_]_*n*_, exists as a linear chain along [111] as the three dinitro­benzoate anions each engages in bridging adjacent dimethyl sulfoxide (DMSO) coordinated Ce^III^ atoms. The metal atoms are surrounded by eight O atoms in a square-anti­prismatic environment. There are two independent formula units in the asymmetric unit. The S atoms of two of the four DMSO mol­ecules are disordered in a 0.926 (3):0.074 (3) ratio.

## Related literature

For isotypic Pr(C_7_H_3_N_2_O_6_)_3_(C_2_H_6_OS)_2_, see: Niu *et al.* (2001 ▶).
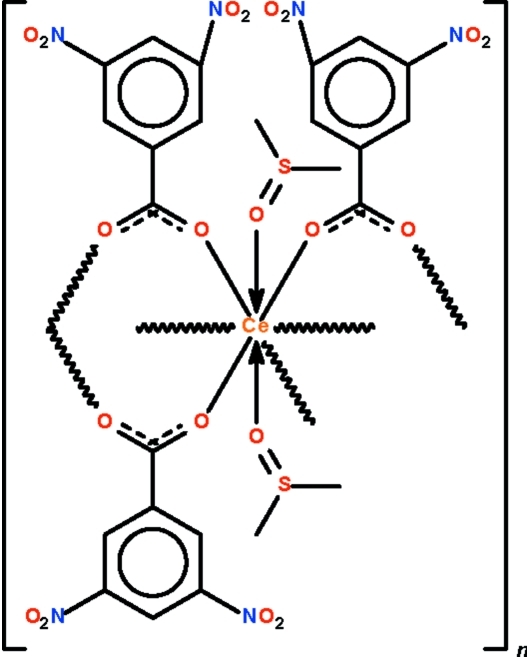


## Experimental

### 

#### Crystal data

[Ce(C_7_H_3_N_2_O_6_)_3_(C_2_H_6_OS)_2_]
*M* *_r_* = 929.72
Triclinic, 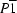

*a* = 14.3025 (10) Å
*b* = 14.3752 (10) Å
*c* = 21.9143 (16) Å
α = 101.8798 (9)°
β = 103.6758 (10)°
γ = 119.6828 (8)°
*V* = 3514.1 (4) Å^3^
*Z* = 4
Mo *K*α radiation
μ = 1.51 mm^−1^
*T* = 293 K
0.25 × 0.20 × 0.15 mm

#### Data collection

Bruker SMART APEX diffractometer
Absorption correction: multi-scan (*SADABS*; Sheldrick, 1996 ▶) *T*_min_ = 0.760, *T*_max_ = 1.000
17917 measured reflections
12456 independent reflections
9611 reflections with *I* > 2σ(*I*)
*R*_int_ = 0.025

#### Refinement

*R*[*F* ^2^ > 2σ(*F* ^2^)] = 0.038
*wR*(*F* ^2^) = 0.099
*S* = 1.03
12456 reflections
980 parameters
6 restraints
H-atom parameters constrained
Δρ_max_ = 0.91 e Å^−3^
Δρ_min_ = −0.69 e Å^−3^

### 

Data collection: *APEX2* (Bruker, 2005 ▶); cell refinement: *SAINT* (Bruker, 2005 ▶); data reduction: *SAINT*; program(s) used to solve structure: *SHELXS97* (Sheldrick, 2008 ▶); program(s) used to refine structure: *SHELXL97* (Sheldrick, 2008 ▶); molecular graphics: *X-SEED* (Barbour, 2001 ▶); software used to prepare material for publication: *publCIF* (Westrip, 2010 ▶).

## Supplementary Material

Crystal structure: contains datablocks global, I. DOI: 10.1107/S1600536811015947/hg5026sup1.cif

Structure factors: contains datablocks I. DOI: 10.1107/S1600536811015947/hg5026Isup2.hkl

Additional supplementary materials:  crystallographic information; 3D view; checkCIF report

## Figures and Tables

**Fig. 1.**
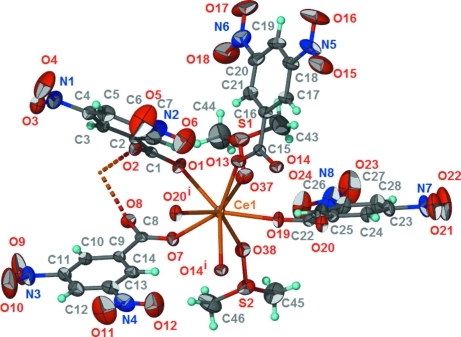
Thermal ellipsoid plot (Barbour, 2001) of a portion of the polymeric chain of Ce(C7H3N2O6)3(C2H6OS)2 at the 50% probability level; hydrogen atoms are drawn as spheres of arbitrary radius. The disorder is not shown. Symmetry code: i = 1 - x, 1 - y, 1 - z.

**Fig. 2.**
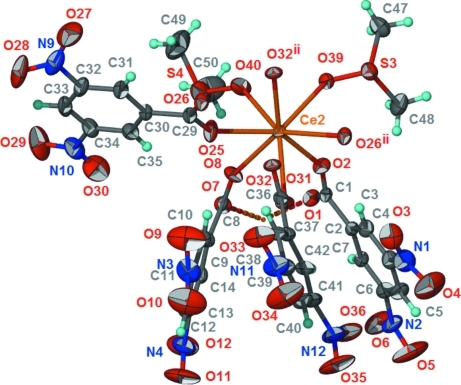
Thermal ellipsoid plot (Barbour, 2001) of a portion of the polymeric chain of Ce(C7H3N2O6)3(C2H6OS)2 at the 50% probability level; hydrogen atoms are drawn as spheres of arbitrary radius. The disorder is not shown. Symmetry code: ii = 2 - x, 2 - y, 2 - z.

**Fig. 3.**
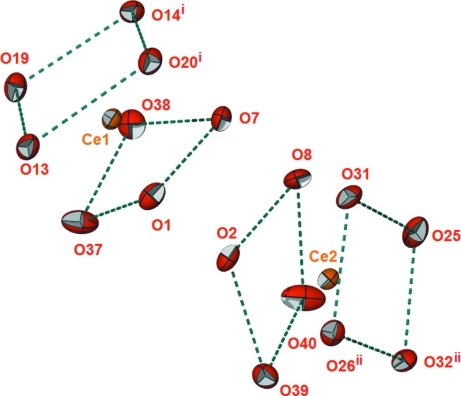
Geometry of Ce atoms.

## supplementary crystallographic information

### Comment

The background to the class of rare-earth 3,5-dinitrobenzoates is given in a report on Pr(C_7_H_3_N_2_O_6_)_3_(C_2_H_6_OS)_2_ (Niu *et al.*, 2001). The DMSO used in the synthesis is incorporated into the crystal structure; DMF in other similar syntheses is also incorporated in the product. The title Ce^III^ analog (Scheme I) is isostructural, the two compounds crystallizing with matching cell dimensions. Polymeric Ce(C_7_H_3_N_2_O_6_)_3_(C_2_H_6_OS)_2_ exists as a linear chain as the three dinitrobenzoate anions each engages in bridging adjacent DMSO-coordinated Ce^III^ atoms (Fig. 1 and Fig. 2). The metal atoms are surrounded by eight O atoms in a square-antiprismatic environment (Fig. 3).

### Experimental

3, 5-Dinitrobenzoic acid (0.434 g) was heated in water (50 ml) at 333 K until it dissolved. Cerium trinitrate hexahydrate (0.442 g) was added followed by dimethyl sulfoxide (4 ml). The solution was heated for 5 h. The solution was filtered and the filtrate evaporated to dryness. The white product was recrystallized from ethanol to furnish colorless primatic crystals.

### Refinement

Carbon-bound H-atoms were placed in calculated positions (C—H 0.93 to 0.97 Å) and were included in the refinement in the riding model approximation, with *U*(H) set to 1.2–1.5*U*(C).

The two DMSO molecules connected to Ce1 are both disordered over two positions in a 93 (1): 7 ratio in respect of the S atoms only. For each molecule, the temperature factors of the primed atom were set to those of the unprimed ones; the anisotropic temperature factors were restranied to be nearly isotropic. Each C–S and C–S' (as well O–S and O–S') pair of bonds were restrained to within 0.01 Å of each other. Their carbon atoms are ordered; however, because of the disordered S atoms, each C atom carries two sets of H-atoms. The ratio of the occupancies is that of the ratio for the S atoms, *i.e.*, 93: 7.

### Crystal data

**Table:**

[Ce(C_7_H_3_N_2_O_6_)_3_(C_2_H_6_OS)_2_]	*Z* = 4
*M**_r_* = 929.72	*F*(000) = 1852
Triclinic, *P*1	*D*_x_ = 1.757 Mg m^−^^3^
Hall symbol: -P 1	Mo *K*α radiation, λ = 0.71073 Å
*a* = 14.3025 (10) Å	Cell parameters from 5527 reflections
*b* = 14.3752 (10) Å	θ = 2.4–24.9°
*c* = 21.9143 (16) Å	µ = 1.51 mm^−^^1^
α = 101.8798 (9)°	*T* = 293 K
β = 103.6758 (10)°	Prism, colorless
γ = 119.6828 (8)°	0.25 × 0.20 × 0.15 mm
*V* = 3514.1 (4) Å^3^	

### Data collection

**Table:**

Bruker SMART APEX diffractometer	12456 independent reflections
Radiation source: fine-focus sealed tube	9611 reflections with *I* > 2σ(*I*)
graphite	*R*_int_ = 0.025
ω scans	θ_max_ = 25.3°, θ_min_ = 1.8°
Absorption correction: multi-scan (*SADABS*; Sheldrick, 1996)	*h* = −17→14
*T*_min_ = 0.760, *T*_max_ = 1.000	*k* = −16→17
17917 measured reflections	*l* = −21→26

### Refinement

**Table:**

Refinement on *F*^2^	Primary atom site location: structure-invariant direct methods
Least-squares matrix: full	Secondary atom site location: difference Fourier map
*R*[*F*^2^ > 2σ(*F*^2^)] = 0.038	Hydrogen site location: inferred from neighbouring sites
*wR*(*F*^2^) = 0.099	H-atom parameters constrained
*S* = 1.03	*w* = 1/[σ^2^(*F*_o_^2^) + (0.0464*P*)^2^ + 1.0209*P*] where *P* = (*F*_o_^2^ + 2*F*_c_^2^)/3
12456 reflections	(Δ/σ)_max_ = 0.001
980 parameters	Δρ_max_ = 0.91 e Å^−^^3^
6 restraints	Δρ_min_ = −0.69 e Å^−^^3^

### Fractional atomic coordinates and isotropic or equivalent isotropic displacement parameters (Å^2^)

**Table:**

	*x*	*y*	*z*	*U*_iso_*/*U*_eq_	Occ. (<1)
Ce1	0.55617 (2)	0.62381 (2)	0.604890 (12)	0.02587 (8)	
Ce2	0.88886 (2)	0.94364 (2)	0.895363 (12)	0.02595 (8)	
S1	0.65997 (14)	0.92117 (12)	0.62797 (10)	0.0595 (6)	0.926 (3)
S2	0.27585 (13)	0.49215 (13)	0.62830 (8)	0.0489 (5)	0.926 (3)
S1'	0.5652 (11)	0.9100 (8)	0.6357 (5)	0.0595 (6)	0.074 (3)
S2'	0.3250 (8)	0.5992 (10)	0.6758 (5)	0.0489 (5)	0.074 (3)
S3	1.03591 (12)	1.22760 (11)	0.87795 (7)	0.0444 (3)	
S4	0.58609 (13)	0.84536 (13)	0.86249 (10)	0.0724 (5)	
O1	0.7610 (3)	0.7927 (3)	0.67902 (15)	0.0387 (8)	
O2	0.8916 (3)	0.9170 (3)	0.78333 (15)	0.0370 (7)	
O3	1.2682 (4)	0.9603 (4)	0.8618 (2)	0.0714 (12)	
O4	1.3286 (4)	0.9145 (5)	0.7871 (3)	0.1074 (19)	
O5	1.0436 (5)	0.6528 (5)	0.5563 (3)	0.126 (2)	
O6	0.8807 (5)	0.6300 (4)	0.5236 (2)	0.0901 (16)	
O7	0.5827 (3)	0.6188 (3)	0.71728 (15)	0.0370 (7)	
O8	0.7161 (3)	0.7416 (2)	0.82119 (16)	0.0423 (8)	
O9	0.8682 (5)	0.5956 (5)	0.9696 (2)	0.112 (2)	
O10	0.8856 (5)	0.4626 (5)	0.9259 (3)	0.121 (2)	
O11	0.6372 (5)	0.2095 (4)	0.6876 (3)	0.111 (2)	
O12	0.5227 (5)	0.2416 (4)	0.6294 (2)	0.0899 (16)	
O13	0.6581 (3)	0.6990 (3)	0.53074 (16)	0.0415 (8)	
O14	0.5787 (3)	0.5885 (2)	0.42033 (15)	0.0354 (7)	
O15	0.7607 (5)	0.7595 (4)	0.2755 (2)	0.0847 (15)	
O16	0.8801 (5)	0.9448 (4)	0.3094 (3)	0.1061 (19)	
O17	1.0087 (4)	1.1887 (4)	0.5405 (3)	0.0972 (18)	
O18	0.9267 (5)	1.1148 (4)	0.6034 (3)	0.1031 (19)	
O19	0.4078 (3)	0.5484 (3)	0.49210 (15)	0.0384 (8)	
O20	0.3154 (3)	0.4520 (3)	0.38036 (16)	0.0375 (8)	
O21	0.0689 (4)	0.5361 (5)	0.2461 (2)	0.0838 (15)	
O22	0.0212 (5)	0.6421 (5)	0.2938 (3)	0.114 (2)	
O23	0.2391 (5)	0.8722 (5)	0.5359 (3)	0.124 (2)	
O24	0.3127 (4)	0.8096 (5)	0.5982 (2)	0.0939 (17)	
O25	0.7997 (3)	0.8387 (3)	0.96524 (17)	0.0490 (9)	
O26	0.8944 (3)	0.9227 (3)	1.07818 (15)	0.0356 (7)	
O27	0.6333 (5)	0.8394 (5)	1.2044 (3)	0.0940 (17)	
O28	0.4968 (4)	0.6564 (5)	1.1708 (3)	0.1001 (18)	
O29	0.2978 (4)	0.4560 (4)	0.9244 (3)	0.1094 (19)	
O30	0.3993 (4)	0.4974 (4)	0.8651 (3)	0.0892 (16)	
O31	0.9693 (3)	0.8182 (3)	0.88109 (16)	0.0375 (8)	
O32	1.0418 (3)	0.9103 (2)	0.99178 (15)	0.0361 (7)	
O33	1.2835 (4)	0.8154 (4)	1.1331 (2)	0.0831 (15)	
O34	1.3600 (5)	0.7397 (6)	1.0878 (3)	0.119 (2)	
O35	1.2544 (5)	0.6112 (5)	0.8471 (3)	0.113 (2)	
O36	1.1658 (5)	0.6658 (4)	0.7884 (2)	0.0875 (15)	
O37	0.5604 (3)	0.8033 (3)	0.6161 (2)	0.0531 (10)	
O38	0.3792 (3)	0.5956 (3)	0.62754 (17)	0.0437 (8)	
O39	0.9244 (3)	1.1215 (3)	0.87207 (16)	0.0384 (8)	
O40	0.7093 (3)	0.9428 (3)	0.8785 (2)	0.0616 (11)	
N1	1.2541 (4)	0.9173 (4)	0.8036 (3)	0.0634 (14)	
N2	0.9755 (5)	0.6729 (4)	0.5663 (3)	0.0619 (14)	
N3	0.8461 (4)	0.5189 (5)	0.9216 (3)	0.0644 (14)	
N4	0.5960 (5)	0.2655 (4)	0.6810 (3)	0.0672 (14)	
N5	0.8163 (5)	0.8577 (5)	0.3180 (3)	0.0695 (15)	
N6	0.9404 (5)	1.1072 (4)	0.5513 (3)	0.0687 (15)	
N7	0.0741 (4)	0.5967 (5)	0.2959 (3)	0.0649 (14)	
N8	0.2656 (5)	0.8066 (5)	0.5436 (3)	0.0751 (17)	
N9	0.5690 (5)	0.7395 (6)	1.1622 (3)	0.0690 (15)	
N10	0.3889 (4)	0.5138 (4)	0.9179 (3)	0.0620 (14)	
N11	1.2961 (5)	0.7739 (5)	1.0839 (3)	0.0664 (14)	
N12	1.2013 (5)	0.6556 (4)	0.8403 (3)	0.0648 (14)	
C1	0.8600 (4)	0.8476 (4)	0.7262 (2)	0.0290 (10)	
C2	0.9502 (4)	0.8311 (4)	0.7113 (2)	0.0287 (10)	
C3	1.0585 (4)	0.8819 (4)	0.7636 (2)	0.0375 (11)	
H3	1.0760	0.9255	0.8076	0.045*	
C4	1.1393 (4)	0.8657 (4)	0.7484 (3)	0.0440 (12)	
C5	1.1166 (5)	0.8000 (5)	0.6848 (3)	0.0518 (14)	
H5	1.1720	0.7897	0.6759	0.062*	
C6	1.0079 (5)	0.7498 (4)	0.6346 (3)	0.0445 (12)	
C7	0.9246 (4)	0.7658 (4)	0.6467 (2)	0.0377 (11)	
H7	0.8528	0.7328	0.6116	0.045*	
C8	0.6579 (4)	0.6469 (4)	0.7738 (2)	0.0291 (10)	
C9	0.6745 (4)	0.5549 (4)	0.7839 (2)	0.0293 (10)	
C10	0.7461 (4)	0.5751 (4)	0.8466 (2)	0.0374 (11)	
H10	0.7787	0.6426	0.8836	0.045*	
C11	0.7690 (4)	0.4947 (4)	0.8543 (3)	0.0418 (12)	
C12	0.7234 (5)	0.3931 (5)	0.8011 (3)	0.0486 (13)	
H12	0.7422	0.3411	0.8064	0.058*	
C13	0.6491 (4)	0.3738 (4)	0.7402 (3)	0.0414 (12)	
C14	0.6235 (4)	0.4514 (4)	0.7301 (2)	0.0382 (11)	
H14	0.5726	0.4347	0.6877	0.046*	
C15	0.6482 (4)	0.6822 (4)	0.4706 (2)	0.0308 (10)	
C16	0.7287 (4)	0.7881 (4)	0.4581 (2)	0.0332 (10)	
C17	0.7315 (4)	0.7759 (4)	0.3945 (2)	0.0374 (11)	
H17	0.6838	0.7034	0.3593	0.045*	
C18	0.8052 (5)	0.8720 (4)	0.3839 (3)	0.0461 (13)	
C19	0.8742 (4)	0.9825 (4)	0.4340 (3)	0.0504 (14)	
H19	0.9218	1.0475	0.4259	0.060*	
C20	0.8684 (4)	0.9905 (4)	0.4967 (3)	0.0451 (13)	
C21	0.7980 (4)	0.8956 (4)	0.5091 (3)	0.0388 (11)	
H21	0.7974	0.9041	0.5522	0.047*	
C22	0.3370 (4)	0.5223 (4)	0.4346 (2)	0.0326 (10)	
C23	0.1498 (4)	0.6191 (5)	0.3641 (3)	0.0438 (12)	
C24	0.2013 (4)	0.5593 (4)	0.3675 (2)	0.0389 (11)	
H24	0.1871	0.5055	0.3283	0.047*	
C25	0.2745 (4)	0.5814 (4)	0.4306 (2)	0.0318 (10)	
C26	0.2942 (4)	0.6602 (4)	0.4889 (2)	0.0392 (11)	
H26	0.3415	0.6738	0.5316	0.047*	
C27	0.2412 (5)	0.7180 (4)	0.4818 (3)	0.0477 (13)	
C28	0.1679 (5)	0.6989 (5)	0.4202 (3)	0.0510 (14)	
H28	0.1324	0.7382	0.4168	0.061*	
C29	0.8052 (4)	0.8515 (4)	1.0240 (2)	0.0327 (10)	
C30	0.6926 (4)	0.7723 (4)	1.0308 (2)	0.0318 (10)	
C31	0.6830 (4)	0.7907 (4)	1.0925 (3)	0.0404 (12)	
H31	0.7472	0.8518	1.1315	0.048*	
C32	0.5784 (5)	0.7186 (5)	1.0962 (3)	0.0473 (13)	
C33	0.4809 (4)	0.6260 (5)	1.0400 (3)	0.0508 (14)	
H33	0.4110	0.5765	1.0435	0.061*	
C34	0.4913 (4)	0.6103 (4)	0.9793 (3)	0.0455 (13)	
C35	0.5951 (4)	0.6810 (4)	0.9731 (2)	0.0395 (11)	
H35	0.5997	0.6678	0.9309	0.047*	
C36	1.0328 (4)	0.8443 (4)	0.9396 (2)	0.0324 (10)	
C37	1.1041 (4)	0.7946 (4)	0.9482 (2)	0.0337 (10)	
C38	1.1634 (4)	0.8043 (4)	1.0115 (2)	0.0388 (11)	
H38	1.1580	0.8395	1.0499	0.047*	
C39	1.2309 (5)	0.7606 (4)	1.0166 (3)	0.0447 (13)	
C40	1.2424 (5)	0.7093 (5)	0.9612 (3)	0.0489 (14)	
H40	1.2882	0.6806	0.9654	0.059*	
C41	1.1835 (5)	0.7026 (4)	0.8998 (3)	0.0476 (13)	
C42	1.1140 (4)	0.7429 (4)	0.8916 (2)	0.0386 (11)	
H42	1.0742	0.7356	0.8487	0.046*	
C43	0.5956 (7)	0.9690 (6)	0.5743 (4)	0.097 (3)	
H43A	0.5727	0.9250	0.5277	0.146*	0.926 (3)
H43B	0.6514	1.0496	0.5848	0.146*	0.926 (3)
H43C	0.5277	0.9582	0.5814	0.146*	0.926 (3)
H43D	0.5649	0.9076	0.5319	0.146*	0.074 (3)
H43E	0.6789	1.0218	0.5889	0.146*	0.074 (3)
H43F	0.5594	1.0095	0.5685	0.146*	0.074 (3)
C44	0.6918 (7)	1.0145 (6)	0.7068 (4)	0.111 (3)	
H44A	0.7284	0.9993	0.7424	0.167*	0.926 (3)
H44B	0.6205	1.0021	0.7090	0.167*	0.926 (3)
H44C	0.7441	1.0934	0.7122	0.167*	0.926 (3)
H44D	0.7491	1.0668	0.6933	0.167*	0.074 (3)
H44E	0.7217	0.9782	0.7291	0.167*	0.074 (3)
H44F	0.6755	1.0568	0.7374	0.167*	0.074 (3)
C45	0.1736 (5)	0.5297 (6)	0.6256 (3)	0.0753 (19)	
H45A	0.1441	0.5307	0.5818	0.113*	0.926 (3)
H45B	0.2120	0.6050	0.6601	0.113*	0.926 (3)
H45C	0.1096	0.4736	0.6337	0.113*	0.926 (3)
H45D	0.1654	0.5422	0.5839	0.113*	0.074 (3)
H45E	0.1418	0.5613	0.6501	0.113*	0.074 (3)
H45F	0.1320	0.4481	0.6158	0.113*	0.074 (3)
C46	0.3100 (7)	0.4971 (6)	0.7116 (3)	0.084 (2)	
H46A	0.3666	0.4791	0.7224	0.126*	0.926 (3)
H46B	0.2401	0.4416	0.7155	0.126*	0.926 (3)
H46C	0.3419	0.5733	0.7426	0.126*	0.926 (3)
H46D	0.2297	0.4312	0.6912	0.126*	0.074 (3)
H46E	0.3329	0.5311	0.7597	0.126*	0.074 (3)
H46F	0.3592	0.4730	0.7035	0.126*	0.074 (3)
C47	0.9983 (5)	1.3264 (5)	0.8680 (3)	0.0576 (15)	
H47A	0.9886	1.3561	0.9077	0.086*	
H47B	0.9263	1.2869	0.8288	0.086*	
H47C	1.0597	1.3893	0.8621	0.086*	
C48	1.0478 (6)	1.1914 (5)	0.7987 (3)	0.0693 (18)	
H48A	1.0681	1.1367	0.7951	0.104*	
H48B	1.1076	1.2602	0.7960	0.104*	
H48C	0.9741	1.1579	0.7622	0.104*	
C49	0.5422 (6)	0.9107 (7)	0.9170 (4)	0.115 (3)	
H49A	0.5828	0.9249	0.9631	0.173*	
H49B	0.4592	0.8597	0.9040	0.173*	
H49C	0.5611	0.9827	0.9131	0.173*	
C50	0.4992 (6)	0.8331 (7)	0.7853 (4)	0.113 (3)	
H50A	0.5109	0.7972	0.7486	0.169*	
H50B	0.5212	0.9088	0.7872	0.169*	
H50C	0.4180	0.7865	0.7782	0.169*	

### Atomic displacement parameters (Å^2^)

**Table:**

	*U*^11^	*U*^22^	*U*^33^	*U*^12^	*U*^13^	*U*^23^
Ce1	0.02805 (14)	0.02672 (14)	0.02143 (14)	0.01626 (12)	0.00806 (11)	0.00719 (11)
Ce2	0.02582 (14)	0.02641 (14)	0.02261 (14)	0.01464 (11)	0.00890 (11)	0.00577 (11)
S1	0.0554 (10)	0.0356 (8)	0.0907 (14)	0.0271 (8)	0.0314 (9)	0.0240 (8)
S2	0.0449 (9)	0.0527 (9)	0.0509 (10)	0.0269 (8)	0.0280 (7)	0.0157 (7)
S1'	0.0554 (10)	0.0356 (8)	0.0907 (14)	0.0271 (8)	0.0314 (9)	0.0240 (8)
S2'	0.0449 (9)	0.0527 (9)	0.0509 (10)	0.0269 (8)	0.0280 (7)	0.0157 (7)
S3	0.0472 (8)	0.0394 (7)	0.0475 (8)	0.0233 (6)	0.0206 (6)	0.0199 (6)
S4	0.0393 (8)	0.0551 (9)	0.1187 (15)	0.0258 (8)	0.0290 (9)	0.0321 (10)
O1	0.0277 (17)	0.0409 (18)	0.0308 (18)	0.0150 (15)	0.0068 (15)	0.0026 (15)
O2	0.0374 (18)	0.0415 (18)	0.0220 (17)	0.0206 (16)	0.0087 (14)	0.0033 (15)
O3	0.053 (3)	0.080 (3)	0.064 (3)	0.034 (2)	0.009 (2)	0.027 (3)
O4	0.054 (3)	0.146 (5)	0.119 (5)	0.069 (3)	0.023 (3)	0.023 (4)
O5	0.107 (4)	0.155 (5)	0.111 (5)	0.094 (4)	0.044 (4)	−0.012 (4)
O6	0.088 (4)	0.108 (4)	0.048 (3)	0.053 (3)	0.026 (3)	−0.006 (3)
O7	0.0425 (19)	0.0360 (17)	0.0228 (17)	0.0192 (16)	0.0079 (15)	0.0094 (14)
O8	0.046 (2)	0.0228 (16)	0.036 (2)	0.0125 (15)	0.0066 (16)	0.0015 (15)
O9	0.171 (6)	0.128 (5)	0.045 (3)	0.111 (5)	0.004 (3)	0.020 (3)
O10	0.134 (5)	0.127 (5)	0.108 (5)	0.102 (4)	−0.001 (4)	0.038 (4)
O11	0.178 (6)	0.080 (3)	0.103 (4)	0.103 (4)	0.043 (4)	0.020 (3)
O12	0.114 (4)	0.069 (3)	0.051 (3)	0.053 (3)	0.004 (3)	−0.006 (2)
O13	0.044 (2)	0.0359 (18)	0.0288 (19)	0.0125 (16)	0.0154 (16)	0.0113 (15)
O14	0.0347 (18)	0.0266 (16)	0.0330 (19)	0.0112 (15)	0.0122 (15)	0.0091 (15)
O15	0.127 (4)	0.078 (3)	0.066 (3)	0.054 (3)	0.065 (3)	0.038 (3)
O16	0.147 (5)	0.084 (3)	0.115 (4)	0.053 (3)	0.098 (4)	0.069 (3)
O17	0.066 (3)	0.035 (2)	0.160 (5)	0.016 (2)	0.037 (3)	0.027 (3)
O18	0.122 (5)	0.045 (3)	0.084 (4)	0.026 (3)	0.030 (4)	−0.006 (3)
O19	0.0353 (18)	0.051 (2)	0.0305 (19)	0.0279 (17)	0.0079 (15)	0.0152 (16)
O20	0.0408 (19)	0.0450 (19)	0.0301 (19)	0.0296 (17)	0.0106 (15)	0.0104 (16)
O21	0.098 (4)	0.140 (4)	0.048 (3)	0.091 (4)	0.026 (3)	0.037 (3)
O22	0.155 (5)	0.182 (6)	0.080 (4)	0.150 (5)	0.031 (3)	0.054 (4)
O23	0.173 (6)	0.121 (4)	0.102 (4)	0.130 (5)	0.019 (4)	0.000 (3)
O24	0.107 (4)	0.127 (4)	0.054 (3)	0.094 (4)	0.014 (3)	−0.005 (3)
O25	0.040 (2)	0.048 (2)	0.033 (2)	0.0081 (17)	0.0180 (16)	0.0110 (16)
O26	0.0278 (17)	0.0361 (17)	0.0325 (19)	0.0127 (15)	0.0111 (15)	0.0110 (15)
O27	0.133 (5)	0.115 (4)	0.066 (3)	0.079 (4)	0.065 (3)	0.041 (3)
O28	0.092 (4)	0.147 (5)	0.111 (4)	0.068 (4)	0.080 (3)	0.091 (4)
O29	0.038 (3)	0.080 (3)	0.134 (5)	−0.005 (3)	0.014 (3)	0.036 (3)
O30	0.068 (3)	0.070 (3)	0.066 (3)	0.020 (3)	0.000 (3)	−0.003 (3)
O31	0.0426 (19)	0.0351 (17)	0.0297 (19)	0.0241 (16)	0.0112 (16)	0.0021 (14)
O32	0.049 (2)	0.0336 (17)	0.0320 (18)	0.0275 (16)	0.0200 (16)	0.0076 (14)
O33	0.129 (4)	0.115 (4)	0.048 (3)	0.099 (4)	0.028 (3)	0.035 (3)
O34	0.152 (5)	0.212 (6)	0.094 (4)	0.162 (5)	0.051 (4)	0.076 (4)
O35	0.176 (5)	0.178 (5)	0.100 (4)	0.162 (5)	0.080 (4)	0.061 (4)
O36	0.152 (5)	0.112 (4)	0.064 (3)	0.104 (4)	0.069 (3)	0.042 (3)
O37	0.047 (2)	0.0344 (19)	0.076 (3)	0.0262 (17)	0.0195 (19)	0.0157 (18)
O38	0.0404 (19)	0.052 (2)	0.046 (2)	0.0280 (18)	0.0226 (17)	0.0205 (17)
O39	0.0429 (19)	0.0369 (18)	0.043 (2)	0.0237 (16)	0.0222 (16)	0.0192 (16)
O40	0.0302 (19)	0.039 (2)	0.108 (3)	0.0208 (17)	0.021 (2)	0.018 (2)
N1	0.043 (3)	0.066 (3)	0.081 (4)	0.035 (3)	0.016 (3)	0.030 (3)
N2	0.064 (3)	0.063 (3)	0.057 (3)	0.036 (3)	0.037 (3)	0.009 (3)
N3	0.062 (3)	0.071 (4)	0.060 (4)	0.041 (3)	0.009 (3)	0.033 (3)
N4	0.098 (4)	0.042 (3)	0.064 (4)	0.042 (3)	0.034 (3)	0.017 (3)
N5	0.095 (4)	0.074 (4)	0.078 (4)	0.052 (4)	0.061 (4)	0.055 (3)
N6	0.057 (3)	0.036 (3)	0.092 (5)	0.024 (3)	0.013 (3)	0.012 (3)
N7	0.071 (3)	0.102 (4)	0.061 (4)	0.070 (3)	0.025 (3)	0.047 (3)
N8	0.077 (4)	0.088 (4)	0.063 (4)	0.067 (4)	0.012 (3)	−0.001 (3)
N9	0.073 (4)	0.110 (5)	0.072 (4)	0.064 (4)	0.050 (3)	0.059 (4)
N10	0.032 (3)	0.043 (3)	0.081 (4)	0.013 (2)	0.005 (3)	0.017 (3)
N11	0.085 (4)	0.091 (4)	0.058 (4)	0.070 (4)	0.027 (3)	0.038 (3)
N12	0.092 (4)	0.075 (4)	0.066 (4)	0.065 (3)	0.048 (3)	0.029 (3)
C1	0.035 (3)	0.032 (2)	0.029 (3)	0.020 (2)	0.019 (2)	0.016 (2)
C2	0.028 (2)	0.029 (2)	0.028 (2)	0.015 (2)	0.013 (2)	0.0097 (19)
C3	0.037 (3)	0.043 (3)	0.037 (3)	0.023 (2)	0.016 (2)	0.019 (2)
C4	0.034 (3)	0.044 (3)	0.054 (3)	0.022 (2)	0.016 (3)	0.023 (3)
C5	0.053 (3)	0.057 (3)	0.069 (4)	0.040 (3)	0.037 (3)	0.028 (3)
C6	0.049 (3)	0.042 (3)	0.045 (3)	0.026 (3)	0.025 (3)	0.014 (2)
C7	0.039 (3)	0.033 (3)	0.041 (3)	0.017 (2)	0.023 (2)	0.013 (2)
C8	0.027 (2)	0.032 (2)	0.029 (3)	0.013 (2)	0.019 (2)	0.014 (2)
C9	0.033 (2)	0.026 (2)	0.027 (2)	0.014 (2)	0.013 (2)	0.0116 (19)
C10	0.035 (3)	0.037 (3)	0.039 (3)	0.019 (2)	0.015 (2)	0.018 (2)
C11	0.043 (3)	0.047 (3)	0.041 (3)	0.026 (3)	0.019 (2)	0.025 (3)
C12	0.053 (3)	0.054 (3)	0.061 (4)	0.038 (3)	0.030 (3)	0.032 (3)
C13	0.054 (3)	0.036 (3)	0.049 (3)	0.029 (3)	0.032 (3)	0.019 (2)
C14	0.043 (3)	0.036 (3)	0.035 (3)	0.019 (2)	0.021 (2)	0.017 (2)
C15	0.032 (2)	0.034 (3)	0.027 (3)	0.017 (2)	0.014 (2)	0.014 (2)
C16	0.038 (3)	0.030 (2)	0.037 (3)	0.021 (2)	0.020 (2)	0.015 (2)
C17	0.040 (3)	0.033 (3)	0.042 (3)	0.019 (2)	0.022 (2)	0.017 (2)
C18	0.050 (3)	0.047 (3)	0.059 (4)	0.030 (3)	0.032 (3)	0.032 (3)
C19	0.041 (3)	0.041 (3)	0.085 (4)	0.024 (3)	0.037 (3)	0.037 (3)
C20	0.033 (3)	0.028 (3)	0.069 (4)	0.016 (2)	0.018 (3)	0.014 (3)
C21	0.041 (3)	0.034 (3)	0.043 (3)	0.023 (2)	0.018 (2)	0.013 (2)
C22	0.027 (2)	0.028 (2)	0.038 (3)	0.015 (2)	0.010 (2)	0.013 (2)
C23	0.044 (3)	0.060 (3)	0.045 (3)	0.037 (3)	0.019 (3)	0.030 (3)
C24	0.044 (3)	0.045 (3)	0.035 (3)	0.030 (3)	0.017 (2)	0.014 (2)
C25	0.030 (2)	0.034 (2)	0.029 (3)	0.018 (2)	0.011 (2)	0.010 (2)
C26	0.036 (3)	0.045 (3)	0.039 (3)	0.027 (2)	0.013 (2)	0.013 (2)
C27	0.053 (3)	0.047 (3)	0.043 (3)	0.034 (3)	0.016 (3)	0.005 (3)
C28	0.058 (3)	0.058 (3)	0.060 (4)	0.049 (3)	0.024 (3)	0.022 (3)
C29	0.029 (3)	0.032 (2)	0.031 (3)	0.014 (2)	0.012 (2)	0.011 (2)
C30	0.029 (2)	0.033 (2)	0.033 (3)	0.016 (2)	0.014 (2)	0.015 (2)
C31	0.035 (3)	0.041 (3)	0.041 (3)	0.018 (2)	0.017 (2)	0.016 (2)
C32	0.052 (3)	0.062 (4)	0.056 (4)	0.038 (3)	0.038 (3)	0.037 (3)
C33	0.036 (3)	0.049 (3)	0.085 (5)	0.026 (3)	0.033 (3)	0.041 (3)
C34	0.026 (3)	0.037 (3)	0.062 (4)	0.014 (2)	0.009 (3)	0.020 (3)
C35	0.038 (3)	0.035 (3)	0.038 (3)	0.019 (2)	0.012 (2)	0.011 (2)
C36	0.032 (3)	0.029 (2)	0.038 (3)	0.019 (2)	0.014 (2)	0.013 (2)
C37	0.037 (3)	0.031 (2)	0.032 (3)	0.021 (2)	0.014 (2)	0.008 (2)
C38	0.055 (3)	0.040 (3)	0.042 (3)	0.036 (3)	0.024 (2)	0.021 (2)
C39	0.053 (3)	0.049 (3)	0.046 (3)	0.037 (3)	0.019 (3)	0.023 (3)
C40	0.059 (3)	0.062 (3)	0.063 (4)	0.051 (3)	0.036 (3)	0.032 (3)
C41	0.064 (4)	0.051 (3)	0.050 (3)	0.043 (3)	0.032 (3)	0.020 (3)
C42	0.050 (3)	0.037 (3)	0.034 (3)	0.029 (2)	0.015 (2)	0.011 (2)
C43	0.119 (7)	0.080 (5)	0.094 (6)	0.054 (5)	0.035 (5)	0.050 (5)
C44	0.137 (7)	0.065 (5)	0.085 (6)	0.053 (5)	0.007 (5)	0.003 (4)
C45	0.065 (4)	0.103 (5)	0.078 (5)	0.057 (4)	0.042 (4)	0.029 (4)
C46	0.118 (6)	0.092 (5)	0.078 (5)	0.064 (5)	0.067 (5)	0.052 (4)
C47	0.065 (4)	0.048 (3)	0.064 (4)	0.034 (3)	0.023 (3)	0.030 (3)
C48	0.080 (5)	0.075 (4)	0.069 (4)	0.041 (4)	0.054 (4)	0.035 (4)
C49	0.069 (5)	0.127 (7)	0.125 (8)	0.042 (5)	0.057 (5)	0.024 (6)
C50	0.079 (5)	0.131 (7)	0.094 (6)	0.066 (6)	0.006 (5)	0.002 (5)

### Geometric parameters (Å, °)

**Table:**

Ce1—O1	2.489 (3)	C3—H3	0.9300
Ce1—O7	2.423 (3)	C4—C5	1.371 (7)
Ce1—O13	2.493 (3)	C5—C6	1.379 (7)
Ce1—O14^i^	2.512 (3)	C5—H5	0.9300
Ce1—O19	2.434 (3)	C6—C7	1.392 (6)
Ce1—O20^i^	2.552 (3)	C7—H7	0.9300
Ce1—O37	2.509 (3)	C8—C9	1.503 (6)
Ce1—O38	2.545 (3)	C9—C10	1.377 (6)
Ce2—O2	2.418 (3)	C9—C14	1.390 (6)
Ce2—O8	2.496 (3)	C10—C11	1.379 (6)
Ce2—O25	2.493 (3)	C10—H10	0.9300
Ce2—O26^ii^	2.535 (3)	C11—C12	1.383 (7)
Ce2—O31	2.582 (3)	C12—C13	1.368 (7)
Ce2—O32^ii^	2.479 (3)	C12—H12	0.9300
Ce2—O39	2.535 (3)	C13—C14	1.377 (6)
Ce2—O40	2.499 (3)	C14—H14	0.9300
S1—O37	1.491 (4)	C15—C16	1.517 (6)
S1—C44	1.749 (7)	C16—C21	1.366 (6)
S1—C43	1.775 (7)	C16—C17	1.381 (6)
S1—H43E	1.7691	C17—C18	1.371 (6)
S1—H44D	1.8212	C17—H17	0.9300
S2—O38	1.497 (3)	C18—C19	1.385 (7)
S2—C46	1.752 (6)	C19—C20	1.380 (8)
S2—C45	1.786 (6)	C19—H19	0.9300
S2—H45F	1.7546	C20—C21	1.368 (6)
S1'—O37	1.468 (9)	C21—H21	0.9300
S1'—C44	1.724 (11)	C22—C25	1.508 (6)
S1'—C43	1.760 (11)	C23—C28	1.368 (7)
S2'—O38	1.458 (9)	C23—C24	1.385 (6)
S2'—C46	1.750 (11)	C24—C25	1.384 (6)
S2'—C45	1.782 (10)	C24—H24	0.9300
S3—O39	1.517 (3)	C25—C26	1.383 (6)
S3—C48	1.781 (5)	C26—C27	1.383 (6)
S3—C47	1.784 (5)	C26—H26	0.9300
S4—O40	1.501 (4)	C27—C28	1.375 (7)
S4—C50	1.761 (8)	C28—H28	0.9300
S4—C49	1.783 (7)	C29—C30	1.511 (6)
O1—C1	1.249 (5)	C30—C31	1.376 (6)
O2—C1	1.240 (5)	C30—C35	1.391 (6)
O3—N1	1.218 (6)	C31—C32	1.369 (7)
O4—N1	1.217 (6)	C31—H31	0.9300
O5—N2	1.199 (6)	C32—C33	1.382 (7)
O6—N2	1.194 (6)	C33—C34	1.359 (7)
O7—C8	1.260 (5)	C33—H33	0.9300
O8—C8	1.229 (5)	C34—C35	1.383 (7)
O9—N3	1.201 (6)	C35—H35	0.9300
O10—N3	1.200 (6)	C36—C37	1.508 (6)
O11—N4	1.222 (6)	C37—C42	1.385 (6)
O12—N4	1.192 (6)	C37—C38	1.382 (6)
O13—C15	1.251 (5)	C38—C39	1.386 (6)
O14—C15	1.250 (5)	C38—H38	0.9300
O14—Ce1^i^	2.512 (3)	C39—C40	1.374 (7)
O15—N5	1.220 (6)	C40—C41	1.364 (7)
O16—N5	1.218 (6)	C40—H40	0.9300
O17—N6	1.213 (7)	C41—C42	1.373 (7)
O18—N6	1.194 (7)	C42—H42	0.9300
O19—C22	1.257 (5)	C43—H43A	0.9600
O20—C22	1.245 (5)	C43—H43B	0.9600
O20—Ce1^i^	2.552 (3)	C43—H43C	0.9600
O21—N7	1.209 (6)	C43—H43D	0.9600
O22—N7	1.219 (6)	C43—H43E	0.9600
O23—N8	1.204 (6)	C43—H43F	0.9600
O24—N8	1.206 (6)	C44—H44A	0.9600
O25—C29	1.240 (5)	C44—H44B	0.9600
O26—C29	1.255 (5)	C44—H44C	0.9600
O26—Ce2^ii^	2.535 (3)	C44—H44D	0.9600
O27—N9	1.227 (7)	C44—H44E	0.9599
O28—N9	1.221 (7)	C44—H44F	0.9599
O29—N10	1.215 (6)	C45—H45A	0.9600
O30—N10	1.192 (6)	C45—H45B	0.9600
O31—C36	1.234 (5)	C45—H45C	0.9600
O32—C36	1.261 (5)	C45—H45D	0.9600
O32—Ce2^ii^	2.479 (3)	C45—H45E	0.9598
O33—N11	1.217 (6)	C45—H45F	0.9600
O34—N11	1.228 (6)	C46—H46A	0.9600
O35—N12	1.214 (6)	C46—H46B	0.9600
O36—N12	1.202 (6)	C46—H46C	0.9600
N1—C4	1.482 (7)	C46—H46D	0.9599
N2—C6	1.471 (7)	C46—H46E	0.9598
N3—C11	1.467 (7)	C46—H46F	0.9600
N4—C13	1.489 (6)	C47—H47A	0.9600
N5—C18	1.473 (7)	C47—H47B	0.9600
N6—C20	1.479 (7)	C47—H47C	0.9600
N7—C23	1.482 (6)	C48—H48A	0.9600
N8—C27	1.484 (7)	C48—H48B	0.9600
N9—C32	1.468 (7)	C48—H48C	0.9600
N10—C34	1.472 (7)	C49—H49A	0.9600
N11—C39	1.462 (7)	C49—H49B	0.9600
N12—C41	1.479 (7)	C49—H49C	0.9600
C1—C2	1.517 (6)	C50—H50A	0.9600
C2—C7	1.373 (6)	C50—H50B	0.9600
C2—C3	1.393 (6)	C50—H50C	0.9600
C3—C4	1.385 (6)		
			
O7—Ce1—O19	143.08 (11)	C13—C12—C11	116.0 (5)
O7—Ce1—O1	75.32 (10)	C13—C12—H12	122.0
O19—Ce1—O1	139.62 (11)	C11—C12—H12	122.0
O7—Ce1—O13	145.04 (11)	C12—C13—C14	123.3 (5)
O19—Ce1—O13	71.83 (10)	C12—C13—N4	118.7 (5)
O1—Ce1—O13	71.68 (10)	C14—C13—N4	117.9 (5)
O7—Ce1—O37	103.30 (12)	C13—C14—C9	119.3 (5)
O19—Ce1—O37	83.09 (11)	C13—C14—H14	120.4
O1—Ce1—O37	72.82 (11)	C9—C14—H14	120.4
O13—Ce1—O37	77.73 (12)	O14—C15—O13	126.8 (4)
O7—Ce1—O14^i^	77.27 (10)	O14—C15—C16	117.4 (4)
O19—Ce1—O14^i^	76.32 (10)	O13—C15—C16	115.7 (4)
O1—Ce1—O14^i^	140.00 (10)	C21—C16—C17	119.7 (4)
O13—Ce1—O14^i^	123.16 (10)	C21—C16—C15	120.6 (4)
O37—Ce1—O14^i^	142.52 (11)	C17—C16—C15	119.7 (4)
O7—Ce1—O38	71.87 (11)	C18—C17—C16	119.3 (5)
O19—Ce1—O38	76.24 (11)	C18—C17—H17	120.4
O1—Ce1—O38	122.96 (10)	C16—C17—H17	120.4
O13—Ce1—O38	137.47 (11)	C17—C18—C19	122.4 (5)
O37—Ce1—O38	71.14 (11)	C17—C18—N5	119.3 (5)
O14^i^—Ce1—O38	73.69 (10)	C19—C18—N5	118.3 (5)
O7—Ce1—O20^i^	77.85 (10)	C20—C19—C18	116.3 (5)
O19—Ce1—O20^i^	118.05 (10)	C20—C19—H19	121.9
O1—Ce1—O20^i^	72.54 (10)	C18—C19—H19	121.9
O13—Ce1—O20^i^	81.56 (11)	C21—C20—C19	122.4 (5)
O37—Ce1—O20^i^	143.73 (11)	C21—C20—N6	119.9 (5)
O14^i^—Ce1—O20^i^	73.63 (10)	C19—C20—N6	117.7 (5)
O38—Ce1—O20^i^	139.33 (10)	C16—C21—C20	119.9 (5)
O2—Ce2—O32^ii^	144.49 (10)	C16—C21—H21	120.0
O2—Ce2—O25	143.39 (11)	C20—C21—H21	120.0
O32^ii^—Ce2—O25	71.96 (10)	O20—C22—O19	124.9 (4)
O2—Ce2—O8	74.83 (11)	O20—C22—C25	117.4 (4)
O32^ii^—Ce2—O8	137.53 (11)	O19—C22—C25	117.7 (4)
O25—Ce2—O8	69.52 (11)	C28—C23—C24	123.0 (5)
O2—Ce2—O40	101.42 (13)	C28—C23—N7	119.2 (5)
O32^ii^—Ce2—O40	81.26 (12)	C24—C23—N7	117.8 (5)
O25—Ce2—O40	76.00 (13)	C23—C24—C25	118.4 (5)
O8—Ce2—O40	72.80 (11)	C23—C24—H24	120.8
O2—Ce2—O39	72.02 (10)	C25—C24—H24	120.8
O32^ii^—Ce2—O39	75.57 (10)	C26—C25—C24	120.7 (4)
O25—Ce2—O39	136.63 (11)	C26—C25—C22	120.4 (4)
O8—Ce2—O39	124.05 (10)	C24—C25—C22	118.8 (4)
O40—Ce2—O39	71.28 (11)	C25—C26—C27	117.9 (5)
O2—Ce2—O26^ii^	77.55 (10)	C25—C26—H26	121.0
O32^ii^—Ce2—O26^ii^	79.01 (10)	C27—C26—H26	121.0
O25—Ce2—O26^ii^	127.03 (10)	C28—C27—C26	123.4 (5)
O8—Ce2—O26^ii^	140.06 (10)	C28—C27—N8	118.0 (5)
O40—Ce2—O26^ii^	141.57 (11)	C26—C27—N8	118.6 (5)
O39—Ce2—O26^ii^	72.02 (10)	C23—C28—C27	116.5 (4)
O2—Ce2—O31	76.18 (10)	C23—C28—H28	121.7
O32^ii^—Ce2—O31	120.76 (10)	C27—C28—H28	121.7
O25—Ce2—O31	85.94 (11)	O25—C29—O26	126.9 (4)
O8—Ce2—O31	73.60 (10)	O25—C29—C30	116.4 (4)
O40—Ce2—O31	145.67 (11)	O26—C29—C30	116.7 (4)
O39—Ce2—O31	136.10 (10)	C31—C30—C35	119.2 (4)
O26^ii^—Ce2—O31	72.17 (10)	C31—C30—C29	121.5 (4)
O32^ii^—Ce2—O32	74.92 (10)	C35—C30—C29	119.3 (4)
O8—Ce2—O32	103.40 (9)	C32—C31—C30	119.6 (5)
O26^ii^—Ce2—O32	65.48 (9)	C32—C31—H31	120.2
O37—S1—C44	106.9 (3)	C30—C31—H31	120.2
O37—S1—C43	104.5 (3)	C31—C32—C33	122.4 (5)
C44—S1—C43	99.1 (4)	C31—C32—N9	119.2 (5)
O38—S2—C46	108.0 (3)	C33—C32—N9	118.4 (5)
O38—S2—C45	103.9 (3)	C34—C33—C32	117.1 (5)
O37—S1'—C44	109.3 (7)	C34—C33—H33	121.4
O37—S1'—C43	106.3 (7)	C32—C33—H33	121.4
C44—S1'—C43	100.6 (6)	C33—C34—C35	122.4 (5)
O38—S2'—C46	110.0 (7)	C33—C34—N10	118.9 (5)
O38—S2'—C45	105.8 (6)	C35—C34—N10	118.7 (5)
C46—S2'—C45	98.0 (6)	C34—C35—C30	119.2 (5)
O39—S3—C48	106.0 (2)	C34—C35—H35	120.4
O39—S3—C47	104.9 (2)	C30—C35—H35	120.4
C48—S3—C47	97.9 (3)	O31—C36—O32	123.8 (4)
O40—S4—C50	105.5 (4)	O31—C36—C37	117.4 (4)
O40—S4—C49	103.3 (3)	O32—C36—C37	118.8 (4)
C50—S4—C49	97.5 (4)	C42—C37—C38	119.9 (4)
C1—O1—Ce1	156.6 (3)	C42—C37—C36	118.8 (4)
C1—O2—Ce2	147.1 (3)	C38—C37—C36	121.3 (4)
C8—O7—Ce1	144.0 (3)	C37—C38—C39	118.8 (5)
C8—O8—Ce2	157.4 (3)	C37—C38—H38	120.6
C15—O13—Ce1	144.5 (3)	C39—C38—H38	120.6
C15—O14—Ce1^i^	139.1 (3)	C40—C39—C38	122.3 (5)
C22—O19—Ce1	172.9 (3)	C40—C39—N11	119.0 (5)
C22—O20—Ce1^i^	113.0 (3)	C38—C39—N11	118.6 (5)
C29—O25—Ce2	143.0 (3)	C41—C40—C39	117.0 (5)
C29—O26—Ce2^ii^	133.4 (3)	C41—C40—H40	121.5
C36—O31—Ce2	104.2 (3)	C39—C40—H40	121.5
C36—O32—Ce2^ii^	165.7 (3)	C40—C41—C42	123.2 (5)
Ce2^ii^—O32—Ce2	105.08 (10)	C40—C41—N12	117.9 (5)
S1'—O37—S1	53.8 (5)	C42—C41—N12	118.8 (5)
S1'—O37—Ce1	169.4 (5)	C41—C42—C37	118.8 (5)
S1—O37—Ce1	128.79 (19)	C41—C42—H42	120.6
S2'—O38—S2	55.1 (5)	C37—C42—H42	120.6
S2'—O38—Ce1	149.0 (4)	S1—C43—H43A	109.5
S2—O38—Ce1	131.60 (19)	S1—C43—H43B	109.5
S3—O39—Ce2	130.77 (18)	H43A—C43—H43B	109.5
S4—O40—Ce2	129.94 (19)	S1—C43—H43C	109.5
O4—N1—O3	124.5 (5)	H43A—C43—H43C	109.5
O4—N1—C4	117.3 (6)	H43B—C43—H43C	109.5
O3—N1—C4	118.2 (5)	S1'—C43—H43D	109.3
O6—N2—O5	122.7 (6)	S1'—C43—H43E	109.6
O6—N2—C6	118.6 (5)	H43D—C43—H43E	109.5
O5—N2—C6	118.6 (6)	S1'—C43—H43F	109.5
O9—N3—O10	123.1 (6)	H43D—C43—H43F	109.5
O9—N3—C11	118.3 (5)	H43E—C43—H43F	109.5
O10—N3—C11	118.6 (6)	S1—C44—H44A	109.5
O12—N4—O11	123.6 (6)	S1—C44—H44B	109.5
O12—N4—C13	119.9 (5)	H44A—C44—H44B	109.5
O11—N4—C13	116.4 (6)	S1—C44—H44C	109.5
O15—N5—O16	124.2 (6)	H44A—C44—H44C	109.5
O15—N5—C18	118.0 (5)	H44B—C44—H44C	109.5
O16—N5—C18	117.9 (6)	S1'—C44—H44D	109.1
O18—N6—O17	124.4 (6)	S1'—C44—H44E	109.4
O18—N6—C20	117.3 (6)	H44D—C44—H44E	109.5
O17—N6—C20	118.3 (6)	S1'—C44—H44F	109.8
O21—N7—O22	123.8 (5)	H44D—C44—H44F	109.5
O21—N7—C23	119.0 (5)	H44E—C44—H44F	109.5
O22—N7—C23	117.2 (5)	S2—C45—H45A	109.5
O24—N8—O23	124.3 (6)	S2—C45—H45B	109.5
O24—N8—C27	117.9 (5)	H45A—C45—H45B	109.5
O23—N8—C27	117.8 (6)	S2—C45—H45C	109.5
O28—N9—O27	124.9 (6)	H45A—C45—H45C	109.5
O28—N9—C32	117.6 (6)	H45B—C45—H45C	109.5
O27—N9—C32	117.5 (5)	S2'—C45—H45D	109.4
O30—N10—O29	123.9 (6)	S2'—C45—H45E	109.6
O30—N10—C34	118.9 (5)	H45D—C45—H45E	109.5
O29—N10—C34	117.2 (6)	S2'—C45—H45F	109.4
O33—N11—O34	122.9 (5)	H45D—C45—H45F	109.5
O33—N11—C39	119.6 (5)	H45E—C45—H45F	109.5
O34—N11—C39	117.6 (5)	S2—C46—H46A	109.5
O36—N12—O35	123.0 (5)	S2—C46—H46B	109.5
O36—N12—C41	119.0 (5)	H46A—C46—H46B	109.5
O35—N12—C41	117.9 (5)	S2—C46—H46C	109.5
O2—C1—O1	125.6 (4)	H46A—C46—H46C	109.5
O2—C1—C2	117.4 (4)	H46B—C46—H46C	109.5
O1—C1—C2	117.0 (4)	S2'—C46—H46D	109.5
C7—C2—C3	120.5 (4)	S2'—C46—H46E	109.5
C7—C2—C1	120.0 (4)	H46D—C46—H46E	109.5
C3—C2—C1	119.5 (4)	S2'—C46—H46F	109.4
C4—C3—C2	118.2 (5)	H46D—C46—H46F	109.5
C4—C3—H3	120.9	H46E—C46—H46F	109.5
C2—C3—H3	120.9	S3—C47—H47A	109.5
C5—C4—C3	123.1 (5)	S3—C47—H47B	109.5
C5—C4—N1	117.8 (5)	H47A—C47—H47B	109.5
C3—C4—N1	119.0 (5)	S3—C47—H47C	109.5
C4—C5—C6	116.9 (5)	H47A—C47—H47C	109.5
C4—C5—H5	121.5	H47B—C47—H47C	109.5
C6—C5—H5	121.5	S3—C48—H48A	109.5
C5—C6—C7	122.3 (5)	S3—C48—H48B	109.5
C5—C6—N2	119.7 (5)	H48A—C48—H48B	109.5
C7—C6—N2	117.9 (5)	S3—C48—H48C	109.5
C2—C7—C6	118.9 (5)	H48A—C48—H48C	109.5
C2—C7—H7	120.6	H48B—C48—H48C	109.5
C6—C7—H7	120.6	S4—C49—H49A	109.5
O8—C8—O7	125.4 (4)	S4—C49—H49B	109.5
O8—C8—C9	118.1 (4)	H49A—C49—H49B	109.5
O7—C8—C9	116.5 (4)	S4—C49—H49C	109.5
C10—C9—C14	118.9 (4)	H49A—C49—H49C	109.5
C10—C9—C8	119.8 (4)	H49B—C49—H49C	109.5
C14—C9—C8	121.2 (4)	S4—C50—H50A	109.5
C9—C10—C11	119.7 (5)	S4—C50—H50B	109.5
C9—C10—H10	120.1	H50A—C50—H50B	109.5
C11—C10—H10	120.1	S4—C50—H50C	109.5
C10—C11—C12	122.7 (5)	H50A—C50—H50C	109.5
C10—C11—N3	118.5 (5)	H50B—C50—H50C	109.5
C12—C11—N3	118.8 (5)		
			
O7—Ce1—O1—C1	37.3 (7)	O9—N3—C11—C10	13.7 (8)
O19—Ce1—O1—C1	−157.4 (7)	O10—N3—C11—C10	−165.0 (6)
O13—Ce1—O1—C1	−131.0 (7)	O9—N3—C11—C12	−166.9 (6)
O37—Ce1—O1—C1	146.5 (7)	O10—N3—C11—C12	14.4 (8)
O14^i^—Ce1—O1—C1	−10.9 (8)	C10—C11—C12—C13	−2.8 (8)
O38—Ce1—O1—C1	93.7 (7)	N3—C11—C12—C13	177.8 (5)
O20^i^—Ce1—O1—C1	−44.4 (7)	C11—C12—C13—C14	2.8 (8)
O32^ii^—Ce2—O2—C1	179.2 (5)	C11—C12—C13—N4	−179.4 (5)
O25—Ce2—O2—C1	−8.1 (6)	O12—N4—C13—C12	173.0 (6)
O8—Ce2—O2—C1	−21.5 (5)	O11—N4—C13—C12	−11.6 (8)
O40—Ce2—O2—C1	−89.9 (5)	O12—N4—C13—C14	−9.1 (8)
O39—Ce2—O2—C1	−155.7 (6)	O11—N4—C13—C14	166.3 (5)
O26^ii^—Ce2—O2—C1	129.4 (5)	C12—C13—C14—C9	−0.3 (7)
O31—Ce2—O2—C1	55.0 (5)	N4—C13—C14—C9	−178.1 (4)
O19—Ce1—O7—C8	169.2 (4)	C10—C9—C14—C13	−2.3 (7)
O1—Ce1—O7—C8	−26.7 (5)	C8—C9—C14—C13	174.4 (4)
O13—Ce1—O7—C8	−7.0 (6)	Ce1^i^—O14—C15—O13	−12.9 (8)
O37—Ce1—O7—C8	−94.6 (5)	Ce1^i^—O14—C15—C16	168.1 (3)
O14^i^—Ce1—O7—C8	123.9 (5)	Ce1—O13—C15—O14	−22.0 (8)
O38—Ce1—O7—C8	−159.3 (5)	Ce1—O13—C15—C16	157.0 (4)
O20^i^—Ce1—O7—C8	48.2 (5)	O14—C15—C16—C21	173.0 (4)
O2—Ce2—O8—C8	31.2 (8)	O13—C15—C16—C21	−6.1 (6)
O32^ii^—Ce2—O8—C8	−166.5 (7)	O14—C15—C16—C17	−6.7 (6)
O25—Ce2—O8—C8	−140.3 (8)	O13—C15—C16—C17	174.2 (4)
O40—Ce2—O8—C8	138.6 (8)	C21—C16—C17—C18	0.8 (7)
O39—Ce2—O8—C8	86.5 (8)	C15—C16—C17—C18	−179.4 (4)
O26^ii^—Ce2—O8—C8	−16.6 (9)	C16—C17—C18—C19	−2.6 (8)
O31—Ce2—O8—C8	−48.6 (8)	C16—C17—C18—N5	174.4 (5)
O7—Ce1—O13—C15	155.5 (5)	O15—N5—C18—C17	−1.6 (8)
O19—Ce1—O13—C15	−22.1 (5)	O16—N5—C18—C17	178.2 (6)
O1—Ce1—O13—C15	175.5 (5)	O15—N5—C18—C19	175.5 (5)
O37—Ce1—O13—C15	−108.8 (5)	O16—N5—C18—C19	−4.7 (8)
O14^i^—Ce1—O13—C15	37.1 (6)	C17—C18—C19—C20	2.4 (8)
O38—Ce1—O13—C15	−65.4 (6)	N5—C18—C19—C20	−174.6 (5)
O20^i^—Ce1—O13—C15	101.2 (5)	C18—C19—C20—C21	−0.5 (8)
O2—Ce2—O25—C29	172.1 (5)	C18—C19—C20—N6	−179.6 (4)
O32^ii^—Ce2—O25—C29	−12.4 (5)	O18—N6—C20—C21	−5.9 (8)
O8—Ce2—O25—C29	−174.1 (6)	O17—N6—C20—C21	174.4 (5)
O40—Ce2—O25—C29	−97.6 (5)	O18—N6—C20—C19	173.1 (6)
O39—Ce2—O25—C29	−55.8 (6)	O17—N6—C20—C19	−6.5 (8)
O26^ii^—Ce2—O25—C29	47.8 (6)	C17—C16—C21—C20	1.0 (7)
O31—Ce2—O25—C29	111.9 (5)	C15—C16—C21—C20	−178.7 (4)
O2—Ce2—O31—C36	151.1 (3)	C19—C20—C21—C16	−1.1 (7)
O32^ii^—Ce2—O31—C36	5.1 (3)	N6—C20—C21—C16	177.9 (4)
O25—Ce2—O31—C36	−61.1 (3)	Ce1^i^—O20—C22—O19	−5.1 (6)
O8—Ce2—O31—C36	−130.9 (3)	Ce1^i^—O20—C22—C25	174.2 (3)
O40—Ce2—O31—C36	−118.8 (3)	O21—N7—C23—C28	171.8 (6)
O39—Ce2—O31—C36	106.7 (3)	O22—N7—C23—C28	−7.6 (8)
O26^ii^—Ce2—O31—C36	70.0 (3)	O21—N7—C23—C24	−6.8 (8)
O32^ii^—Ce2—O32—C36	−170.3 (3)	O22—N7—C23—C24	173.9 (6)
O8—Ce2—O32—C36	53.6 (3)	C28—C23—C24—C25	−0.1 (8)
O26^ii^—Ce2—O32—C36	−85.7 (3)	N7—C23—C24—C25	178.5 (4)
O2—Ce2—O32—Ce2^ii^	144.15 (10)	C23—C24—C25—C26	1.0 (7)
O32^ii^—Ce2—O32—Ce2^ii^	0.0	C23—C24—C25—C22	−176.2 (4)
O25—Ce2—O32—Ce2^ii^	−76.85 (12)	O20—C22—C25—C26	179.8 (4)
O8—Ce2—O32—Ce2^ii^	−136.15 (11)	O19—C22—C25—C26	−0.9 (6)
O40—Ce2—O32—Ce2^ii^	−57.08 (18)	O20—C22—C25—C24	−3.0 (6)
O39—Ce2—O32—Ce2^ii^	54.11 (16)	O19—C22—C25—C24	176.3 (4)
O26^ii^—Ce2—O32—Ce2^ii^	84.59 (12)	C24—C25—C26—C27	−1.8 (7)
O31—Ce2—O32—Ce2^ii^	172.95 (18)	C22—C25—C26—C27	175.3 (4)
C44—S1—O37—Ce1	−116.4 (4)	C25—C26—C27—C28	1.8 (8)
C43—S1—O37—Ce1	139.2 (3)	C25—C26—C27—N8	−177.3 (5)
O7—Ce1—O37—S1	98.3 (3)	O24—N8—C27—C28	167.5 (6)
O19—Ce1—O37—S1	−118.6 (3)	O23—N8—C27—C28	−13.9 (9)
O1—Ce1—O37—S1	28.6 (3)	O24—N8—C27—C26	−13.3 (9)
O13—Ce1—O37—S1	−45.8 (3)	O23—N8—C27—C26	165.3 (6)
O14^i^—Ce1—O37—S1	−175.3 (2)	C24—C23—C28—C27	0.1 (8)
O38—Ce1—O37—S1	163.6 (3)	N7—C23—C28—C27	−178.4 (5)
O20^i^—Ce1—O37—S1	10.8 (4)	C26—C27—C28—C23	−1.0 (8)
C46—S2—O38—Ce1	91.7 (3)	N8—C27—C28—C23	178.2 (5)
C45—S2—O38—Ce1	−165.1 (3)	Ce2—O25—C29—O26	−29.4 (9)
O7—Ce1—O38—S2'	14.8 (9)	Ce2—O25—C29—C30	151.1 (4)
O19—Ce1—O38—S2'	175.9 (10)	Ce2^ii^—O26—C29—O25	−10.2 (7)
O1—Ce1—O38—S2'	−43.2 (10)	Ce2^ii^—O26—C29—C30	169.4 (3)
O13—Ce1—O38—S2'	−142.0 (9)	O25—C29—C30—C31	−169.1 (4)
O37—Ce1—O38—S2'	−96.8 (10)	O26—C29—C30—C31	11.3 (6)
O14^i^—Ce1—O38—S2'	96.4 (10)	O25—C29—C30—C35	8.6 (6)
O20^i^—Ce1—O38—S2'	58.7 (10)	O26—C29—C30—C35	−171.0 (4)
O7—Ce1—O38—S2	−74.8 (3)	C35—C30—C31—C32	0.6 (7)
O19—Ce1—O38—S2	86.4 (3)	C29—C30—C31—C32	178.3 (4)
O1—Ce1—O38—S2	−132.8 (2)	C30—C31—C32—C33	0.7 (8)
O13—Ce1—O38—S2	128.5 (2)	C30—C31—C32—N9	−179.6 (5)
O37—Ce1—O38—S2	173.6 (3)	O28—N9—C32—C31	−152.9 (5)
O14^i^—Ce1—O38—S2	6.8 (2)	O27—N9—C32—C31	27.8 (8)
O20^i^—Ce1—O38—S2	−30.9 (3)	O28—N9—C32—C33	26.8 (8)
C48—S3—O39—Ce2	87.1 (3)	O27—N9—C32—C33	−152.4 (6)
C47—S3—O39—Ce2	−170.0 (3)	C31—C32—C33—C34	−1.7 (8)
O2—Ce2—O39—S3	−81.3 (2)	N9—C32—C33—C34	178.5 (5)
O32^ii^—Ce2—O39—S3	84.0 (2)	C32—C33—C34—C35	1.6 (8)
O25—Ce2—O39—S3	126.4 (2)	C32—C33—C34—N10	−179.0 (4)
O8—Ce2—O39—S3	−137.8 (2)	O30—N10—C34—C33	−177.2 (6)
O40—Ce2—O39—S3	169.5 (3)	O29—N10—C34—C33	3.4 (8)
O26^ii^—Ce2—O39—S3	1.1 (2)	O30—N10—C34—C35	2.2 (8)
O31—Ce2—O39—S3	−35.6 (3)	O29—N10—C34—C35	−177.2 (5)
C50—S4—O40—Ce2	−121.1 (4)	C33—C34—C35—C30	−0.4 (7)
C49—S4—O40—Ce2	137.1 (4)	N10—C34—C35—C30	−179.8 (4)
O2—Ce2—O40—S4	95.7 (3)	C31—C30—C35—C34	−0.7 (7)
O32^ii^—Ce2—O40—S4	−120.2 (3)	C29—C30—C35—C34	−178.5 (4)
O25—Ce2—O40—S4	−46.8 (3)	Ce2—O31—C36—O32	5.7 (5)
O8—Ce2—O40—S4	25.7 (3)	Ce2—O31—C36—C37	−173.6 (3)
O39—Ce2—O40—S4	162.1 (4)	Ce2^ii^—O32—C36—O31	−143.5 (9)
O26^ii^—Ce2—O40—S4	−180.0 (2)	Ce2^ii^—O32—C36—C37	35.8 (14)
O31—Ce2—O40—S4	13.6 (5)	O31—C36—C37—C42	12.9 (6)
Ce2—O2—C1—O1	79.9 (7)	O32—C36—C37—C42	−166.5 (4)
Ce2—O2—C1—C2	−102.5 (5)	O31—C36—C37—C38	−170.0 (4)
Ce1—O1—C1—O2	−98.1 (8)	O32—C36—C37—C38	10.7 (7)
Ce1—O1—C1—C2	84.3 (8)	C42—C37—C38—C39	−0.6 (7)
O2—C1—C2—C7	−172.6 (4)	C36—C37—C38—C39	−177.7 (4)
O1—C1—C2—C7	5.2 (6)	C37—C38—C39—C40	0.9 (8)
O2—C1—C2—C3	7.9 (6)	C37—C38—C39—N11	178.3 (5)
O1—C1—C2—C3	−174.3 (4)	O33—N11—C39—C40	−177.4 (6)
C7—C2—C3—C4	0.7 (7)	O34—N11—C39—C40	1.0 (9)
C1—C2—C3—C4	−179.7 (4)	O33—N11—C39—C38	5.1 (8)
C2—C3—C4—C5	−1.6 (7)	O34—N11—C39—C38	−176.5 (6)
C2—C3—C4—N1	−178.9 (4)	C38—C39—C40—C41	−0.2 (8)
O4—N1—C4—C5	11.9 (8)	N11—C39—C40—C41	−177.6 (5)
O3—N1—C4—C5	−168.8 (5)	C39—C40—C41—C42	−0.8 (8)
O4—N1—C4—C3	−170.6 (5)	C39—C40—C41—N12	175.6 (5)
O3—N1—C4—C3	8.6 (7)	O36—N12—C41—C40	−168.2 (6)
C3—C4—C5—C6	0.7 (8)	O35—N12—C41—C40	9.7 (8)
N1—C4—C5—C6	178.0 (5)	O36—N12—C41—C42	8.3 (8)
C4—C5—C6—C7	1.1 (8)	O35—N12—C41—C42	−173.7 (6)
C4—C5—C6—N2	−176.6 (5)	C40—C41—C42—C37	1.0 (8)
O6—N2—C6—C5	179.7 (6)	N12—C41—C42—C37	−175.4 (5)
O5—N2—C6—C5	2.3 (8)	C38—C37—C42—C41	−0.3 (7)
O6—N2—C6—C7	1.9 (8)	C36—C37—C42—C41	176.9 (4)
O5—N2—C6—C7	−175.5 (6)	O37—S1'—C43—S1	−52.5 (5)
C3—C2—C7—C6	0.9 (7)	C44—S1'—C43—S1	61.4 (5)
C1—C2—C7—C6	−178.6 (4)	O37—S1—C43—S1'	50.8 (5)
C5—C6—C7—C2	−1.9 (7)	C44—S1—C43—S1'	−59.4 (4)
N2—C6—C7—C2	175.8 (4)	O37—S1'—C44—S1	50.2 (6)
Ce2—O8—C8—O7	−96.2 (8)	C43—S1'—C44—S1	−61.4 (5)
Ce2—O8—C8—C9	85.4 (8)	O37—S1—C44—S1'	−48.3 (5)
Ce1—O7—C8—O8	86.0 (6)	C43—S1—C44—S1'	60.0 (5)
Ce1—O7—C8—C9	−95.6 (5)	O38—S2'—C45—S2	52.3 (5)
O8—C8—C9—C10	5.2 (6)	C46—S2'—C45—S2	−61.1 (4)
O7—C8—C9—C10	−173.4 (4)	O38—S2—C45—S2'	−49.9 (4)
O8—C8—C9—C14	−171.6 (4)	C46—S2—C45—S2'	60.9 (4)
O7—C8—C9—C14	9.9 (6)	O38—S2'—C46—S2	−48.6 (5)
C14—C9—C10—C11	2.3 (7)	C45—S2'—C46—S2	61.5 (4)
C8—C9—C10—C11	−174.5 (4)	O38—S2—C46—S2'	46.2 (4)
C9—C10—C11—C12	0.4 (7)	C45—S2—C46—S2'	−61.2 (4)
C9—C10—C11—N3	179.7 (4)		

Symmetry codes: (i) −*x*+1, −*y*+1, −*z*+1; (ii) −*x*+2, −*y*+2, −*z*+2.
